# Antileukemic Potential of Sodium Caseinate in Cytarabine-Resistant HL60-CR50 Human Leukemia Cells

**DOI:** 10.3390/molecules30244759

**Published:** 2025-12-12

**Authors:** Edelmiro Santiago-Osorio, Daniel Romero-Trejo, Víctor Manuel Macías-Zaragoza, Katia Michell Rodríguez-Terán, Víctor Manuel Mendoza-Núñez, Edith Sierra-Mondragón, Ernesto Romero-López, Lorena Shira, David Hernández-Álvarez, Itzen Aguiñiga-Sánchez

**Affiliations:** 1 Hematopoiesis and Leukemia Laboratory, Research Unit on Cell Differentiation and Cancer, Faculty of High Studies Zaragoza, National Autonomous University of Mexico, Mexico City 09230, Mexico; edelmiro@unam.mx (E.S.-O.); danielromerot19@gmail.com (D.R.-T.); iram66671@yahoo.es (V.M.M.-Z.); katia.rodriguez.0199@gmail.com (K.M.R.-T.); dr.ernestoromerolopez@gmail.com (E.R.-L.); lorena@immunotherapymx.com (L.S.); 2 Department of Biomedical Sciences, School of Medicine, Faculty of High Studies Zaragoza, National Autonomous University of Mexico, Mexico City 56410, Mexico; 3 Research Unit on Gerontology, Faculty of High Studies Zaragoza, National Autonomous University of Mexico, Mexico City 09230, Mexico; mendibig@unam.mx (V.M.M.-N.); hereda2912@gmail.com (D.H.-Á.); 4 Department of Physiology, Biophysics, and Neurosciences, Center for Research and Advanced Studies of the National Polytechnic Institute, Mexico City 07360, Mexico; edith_fisiologiacelular@outlook.com

**Keywords:** leukemia, cytarabine, chemoresistance, milk protein, sodium caseinate, apoptosis

## Abstract

Chemoresistance is the leading cause of mortality in cancer patients. The poor clinical prognosis and limited therapeutic options for acute myeloid leukemia (AML) patients demand the development of new therapeutic strategies capable of overcoming chemoresistance and avoiding toxic side effects in normal cells. Sodium caseinate (SC), a derivative of casein protein found in milk, has demonstrated a dual role: it inhibits the proliferation of several murine AML cell lines while promoting the proliferation of normal hematopoietic cells. Furthermore, we previously showed that SC can modulate the expression of genes associated with chemoresistance in mouse cells. However, its biological effects on cytarabine-resistant human leukemia cells remain unclear. Here, we developed the HL60-CR50 subline, resistant to cytarabine, and investigated the effects of SC. We demonstrated that SC significantly reduced cell proliferation, decreased SIRT1 levels, increased acetylated p53, activated cleaved caspase-3, and enhanced apoptosis in cytarabine-resistant cells. These findings suggest that SC might have potential as a therapeutic adjuvant for AML, providing efficacy in chemoresistant cases compared with cytarabine treatment alone.

## 1. Introduction

Cytarabine (1-β-D-arabinofuranosylcytosine; Ara-C) is the key chemotherapeutic agent for treatment of acute myeloid leukemia (AML) [1]. The standard induction chemotherapy has been used for more than 50 years as the gold standard [2,3], which consists of conventional doses of 7-day administration of Ara-C plus 3-day administration of anthracycline (daunorubicin, idarubicin) and provides over 70% remission rates for AML [4,5,6]. Nevertheless, only 30% of the patients are long-term survivors; most deteriorate, relapse, and do not respond to further chemotherapy regimens due to the development of resistance to Ara-C [7]. Despite the availability of complementary therapies such as the liposomal combination of Ara-C and daunorubicin (CPX-351), all-trans retinoic acid (ATRA), arsenic trioxide, and various selective inhibitors, these approaches remain insufficient to fully eradicate leukemic cells due to relapse and the development of chemoresistance [6,8,9]. Therefore, there is an urgent need to develop selective therapies capable of eliminating leukemic cells while minimizing toxic side effects on healthy tissues.

Although the mechanisms underlying chemoresistance in AML have not been fully elucidated, substantial evidence indicates that decreased deoxycytidine kinase (dCK) activity and reduced expression of the human equilibrative nucleoside transporter 1 (hENT1) are major determinants of cytarabine resistance in AML patients and leukemia-derived sublines [10,11,12,13]. Additionally, the overexpression of SIRT1, a deacetylase protein, has emerged as a key regulator of tumor cell persistence and resistance to chemotherapeutic agents through its modulation of tumor suppressor pathways involving p53 and PTEN, ultimately inhibiting apoptosis [14,15,16,17]. Consequently, therapeutic strategies capable of circumventing or reversing these resistance mechanisms hold considerable promise for improving clinical outcomes.

The exploration of bioactive natural compounds as potential anticancer agents has gained considerable attention; however, the antitumor properties of milk-derived proteins remain relatively underexplored. Sodium caseinate (SC), a salt derived from casein, the predominant protein in milk, can suppress proliferation and promote apoptosis in murine AML cells, as well as extend survival in leukemia-bearing mice [15,18,19]. In addition, SC stimulates the in vitro proliferation of normal mononuclear cells from BALB/c mice, indicating a selective inhibitory activity against AML cells without harming healthy cells [18,19,20]. Moreover, we have demonstrated that SC regulates the expression of genes involved in cytarabine resistance in mouse AML models. Despite this knowledge gap, it is still unclear whether the antileukemic properties of SC persist in human leukemic cells that have acquired resistance to cytarabine. In the present study, we generated a cytarabine-resistant human leukemia subline derived from HL-60 cells (HL60-CR50) to mimic the resistant phenotype observed in patients who do not respond to therapy due to acquired resistance [21]. We demonstrate that SC exhibits antileukemic activity by inhibiting cell proliferation through the downregulation of SIRT1, which in turn increases p53 acetylation and enhances caspase-3 activation, ultimately promoting apoptosis.

## 2. Results

### 2.1. Development of a Cytarabine-Resistant HL-60 Human Cell Line

**Figure 1:**
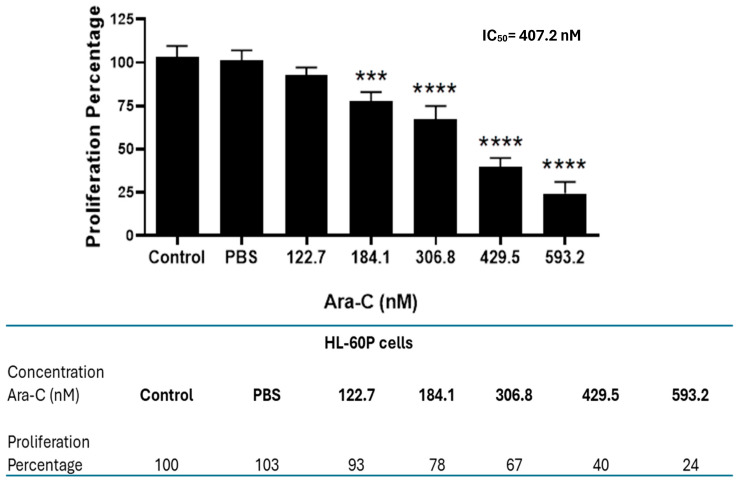
Cytarabine induces cytotoxicity in parental HL-60P cells. The cytotoxic effects of Ara-C on parental HL-60P cells were evaluated using the crystal violet assay with increasing concentrations of Ara-C after 48 h of incubation in the presence or absence of treatment. The IC_50_ value of Ara-C in parental cells was 407.2 nM. Data were compared to the control group and PBS. Results shown are mean ± SD of three independent experiments, each with triplicate samples. One-way ANOVA, followed by Tukey’s, *** *p* < 0.001, **** *p* < 0.0001.

**Figure 2:**
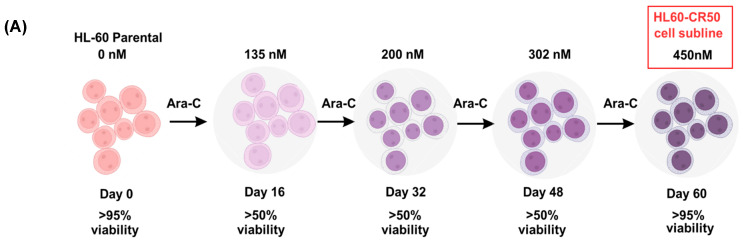

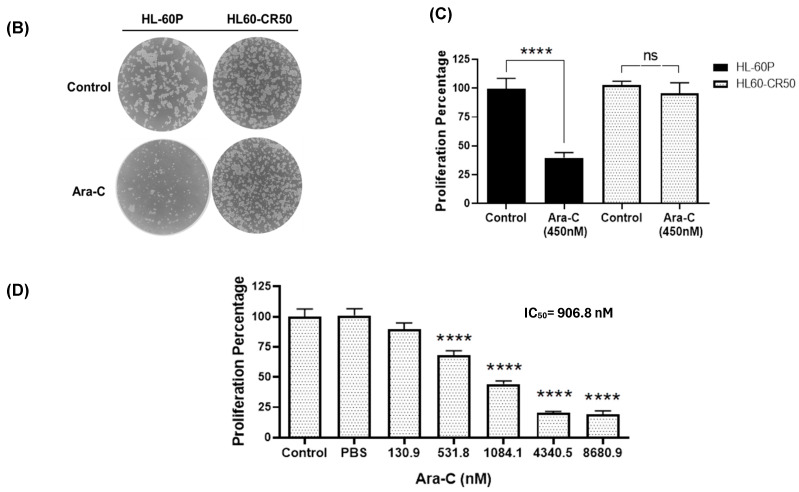
Establishment of a cytarabine-resistant HL60-CR50 cell subline. (**A**) Parental HL-60 cells were cultured with increasing concentrations of Ara-C, starting at 135 nM and gradually escalating to a final concentration of 450 nM. (**B**) Representative photographs of parental HL-60P and HL60-CR50 cells treated with or without 450 nM Ara-C. (**C**) Percentage of proliferation of parental and resistant cells treated in the presence or absence of 450 nM cytarabine. (**D**) Percentage of proliferation in HL60-CR50 cells treated with high concentrations of Ara-C. Data are presented as mean ± SD from three independent experiments, each performed in triplicate. Statistical analysis was conducted using student test or one-way ANOVA followed by Tukey’s post hoc test; **** *p* < 0.0001, ns, non-significant.

As a first approach to generating a cytarabine-resistant cell subline, a proliferation assay was performed using parental HL-60 cells exposed to increasing concentrations of Ara-C. A clear dose-dependent cytotoxic effect was observed, with a calculated IC_50_ value of 407.2 nM for Ara-C (Figure 1). Based on these results, an HL-60 subline resistant to Ara-C (HL60-CR50) was generated from the parental HL-60 cells. Initially, the culture was started with 135 nM of Ara-C, with incremental increases every 16 days, until reaching a final concentration of 450 nM of Ara-C (Figure 2A). Using a cell proliferation assay, we demonstrated that HL60-CR50 cells, in the presence or absence of 450 nM of Ara-C, did not show significant changes in cell number (Figure 2B) or percentage of cell proliferation compared to parental HL-60 cells treated with Ara-C (Figure 2C). Additionally, HL60-CR50 cells treated with Ara-C required higher concentrations to achieve a comparable reduction in proliferation (Figure 2D) compared with parental HL-60 cells (Figure 1), indicating cytarabine resistance. These results support the generation of an HL-60 human subline resistant to Ara-C.

### 2.2. Molecular Characterization of Cytarabine-Resistant HL60-CR50 Cells

**Figure 3:**
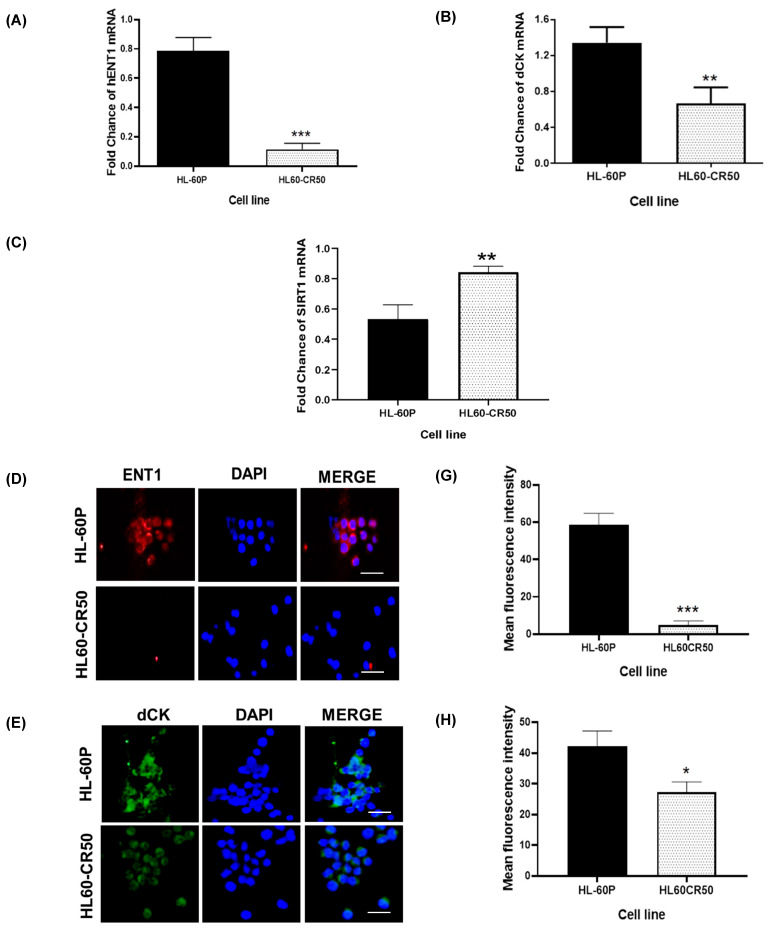

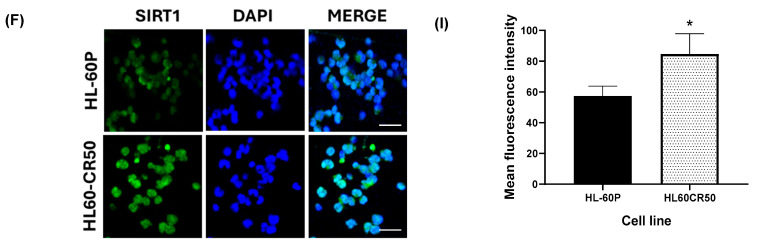
Molecular characterization of parental HL-60 and cytarabine-resistant HL60-CR50 cells. (**A**–**C**) Real-time RT-PCR analysis of hENT1, dCK, and SIRT1 expression (data represent the fold-change mean value of mRNA expression from three independent measurements). (**D**–**F**) Representative confocal microscopy images showing hENT1, dCK and SIRT1 expression, respectively: staining for hENT1, dCK and SIRT1 (**left panel**); nuclei stained with DAPI (**middle panel**); and merged images (**right panel**) in parental and resistant cells. Scale bar: 50 μm. (**G**–**I**) Quantification of hENT1, dCK, and SIRT1 proteins. Fluorescence intensity was measured for individual cells within three randomly selected fields per experimental condition, with 100–120 cells analyzed per treatment group. Bars represent mean ± SD from three independent experiments. Statistical analysis was conducted using student test; * *p* < 0.1; ** *p* < 0.01, *** *p* < 0.001.

To validate the acquisition of chemoresistance in HL60-CR50 cells, we examined the expression of deoxycytidine kinase (dCK) and, equilibrative nucleoside transporter 1 (ENT1) two essential molecules involved in the intracellular uptake and metabolic activation of Ara-C [10,11,22,23]. We also assessed the expression of SIRT1, an oncogenic gene associated with chemoresistance [15]. Using RT-qPCR and immunofluorescence analyses, we observed that HL60-CR50 cells exhibited markedly reduced levels of hENT1 and dCK, together with an increased expression of SIRT1, relative to the parental HL-60 line (Figure 3A–I). Collectively, these findings confirm the successful generation of the Ara-C–resistant HL60-CR50 subline and validate its suitability as a model to investigate the effects of SC on cytarabine-resistant leukemia cells.

### 2.3. Sodium Caseinate Exerts Antiproliferative and Pro-Apoptotic Effects in HL60-CR50 Cells

**Figure 4:**
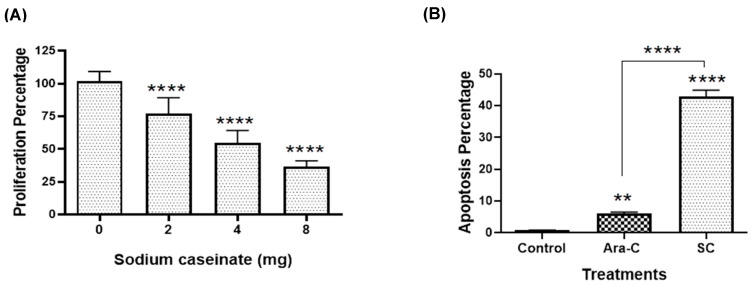

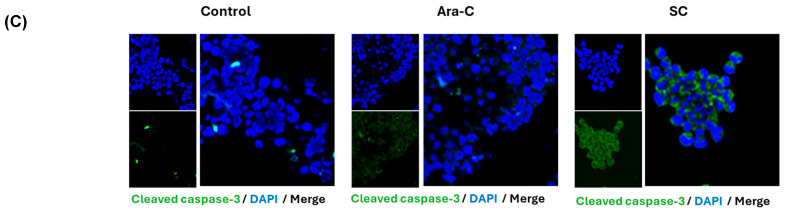
Sodium caseinate induces cytotoxicity, apoptosis and cleaved caspase-3 activation in HL60-CR50 cells. (**A**) Cytotoxic effect of SC on HL60-CR50 cells evaluated by crystal violet assay. (**B**) The percentage of total apoptosis (including both early and late stages) was measured in HL60-CR50 cells treated with or without SC (5.7 mg/mL) or Ara-C (450 nM) for 72 h, using the Annexin V/7-AAD assay. (**C**) Immunofluorescence analysis shows that SC promotes cleaved caspase-3 activation: staining for cleaved caspase-3 (**bottom left panel**); nuclei stained with DAPI (**top left panel**); and overlay images (**right panel**) in resistant cells. Scale bar: 50 μm. Data are presented as mean ± SD from three independent experiments, each performed in triplicate. Statistical analysis was performed using one-way ANOVA followed by Tukey’s post hoc test; ** *p* < 0.01, **** *p* < 0.0001.

As a first study to evaluate the effect of SC on the proliferation of chemoresistant promyelocytic cells, we performed cell proliferation assays. As shown in Figure 4A, SC showed a concentration-dependent cytotoxic effect, with an IC_50_ value of 5.7 mg/mL. Furthermore, apoptosis analysis using annexin V and 7-AAD staining followed by flow cytometry revealed that SC significantly increased apoptosis by approximately 45% compared with the PBS or Ara-C (450 nM) groups (Figure 4B). In addition, immunofluorescence analysis revealed a marked increase in cleaved caspase-3 levels in HL60-CR50 cells treated with SC compared with the other experimental groups (Figure 4C). Collectively, these results indicate that SC exerts a cytotoxic effect on cytarabine-resistant cells by reducing cell proliferation and promoting apoptosis through the activation of cleaved caspase-3.

### 2.4. Sodium Caseinate Promotes p53 Acetylation by Decreasing SIRT1 Expression

**Figure 5:**
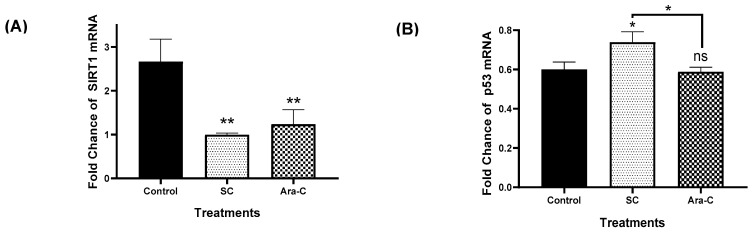

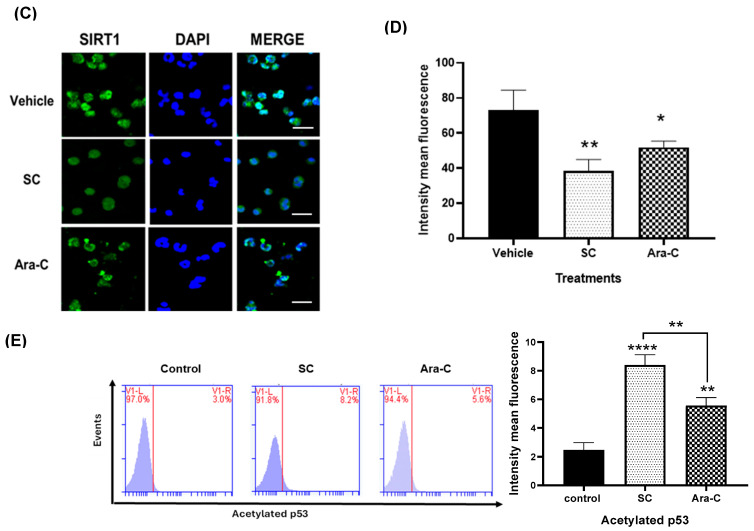
Sodium caseinate decreases SIRT1 levels, allowing p53 acetylation in cytarabine-resistant cells. (**A**,**B**) Real-time RT-PCR analysis of SIRT1, and p53 expression (data represent the foldchange mean value of mRNA expression from three independent measurements). (**C**) Immunofluorescence images show that SC decreases SIRT1 expression levels. Scale bar: 50 μm. (**D**) SIRT1 protein levels were quantified by measuring fluorescence intensity in individual cells from three randomly selected fields per experimental condition. A total of 100–120 cells were analyzed for each treatment group, and results are presented as mean ± SD from three independent experiments. (**E**) Effect of SC (5.7 mg/mL) or Ara-C (450 nM) on acetylated p53 levels in HL60-CR50 cells. Statistical analysis was conducted using one-way ANOVA followed by Tukey’s post hoc test; * *p* < 0.1; ** *p* < 0.01, **** *p* < 0.0001, ns, non-significant.

The deacetylation of *p53* at lysine 382 by SIRT1 decreases *p53*-dependent apoptosis in response to DNA damage in human non-small cell lung cancer (NCI-H1299), human breast cancer (MCF-7L), and acute myeloid leukemia (AML) cells, thereby promoting chemoresistance and cancer cell proliferation [6,17,24]. In this study, we demonstrated by RT-qPCR and immunofluorescence that both SC and Ara-C significantly reduced SIRT1 expression compared with the control group (Figure 5A,D). No significant differences in SIRT1 levels were detected between the SC- and Ara-C-treated groups (Figure 5A,D). However, RT-qPCR analysis showed that SC treatment markedly increased p53 expression relative to both the Ara-C (450 nM) and control groups (Figure 5B). Additionally, flow cytometry revealed that SC treatment led to a significant increase in acetylated p53 compared with the Ara-C-treated and control groups (Figure 5E). These results suggest that SC not only exerts antiproliferative and pro-apoptotic effects on human cytarabine-resistant cells, but also downregulates key genes involved in chemoresistance, such as SIRT1, thereby enhancing the acetylation of the tumor suppressor p53 and potentially providing a therapeutic advantage over conventional treatment.

## 3. Discussion

Tumor cells employ a wide range of mechanisms to develop chemoresistance and evade drug-induced cytotoxicity, including increased drug efflux mediated by ATP-binding cassette (ABC) transporters, reduced intracellular drug uptake, alterations in drug metabolism, inhibition of apoptosis, and activation of pro-survival signaling pathways [21,25,26,27]. Therefore, the development of a cytarabine-resistant cell line represents a valuable experimental strategy for studying hematologic malignancies, facilitating a comprehensive investigation of the molecular mechanisms underlying cytotoxicity and chemoresistance [13,28,29]. In the present study, we successfully established a cytarabine-resistant human leukemia subline, designated HL60-CR50, derived from the parental HL-60 cell line, and evaluated the cellular responses to sodium caseinate (SC), a potential antileukemic agent. Our observations showed that, in resistant cells, the IC_50_ value for cytarabine was 906.8 nM, approximately 2.2-fold higher than that observed in the parental HL-60 cells (407.2 nM). This indicates that a higher concentration of cytarabine is required to achieve complete cytotoxicity in the resistant subline compared to the parental cells. This finding is consistent with previous reports showing that chemoresistant AML cells are capable of proliferating even in the presence of Ara-C [13,21,30,31].

In agreement with clinical observations, patients with AML frequently relapse after chemotherapy, and mutations or alterations in hENT1 and dCK are among the most common findings in AML patient samples [24] and cell lines [13,32,33,34]. In addition, the overexpression of SIRT1 has been shown to contribute to chemoresistance in various cancer types, including AML [15,35,36,37], by promoting the activation of ERK1/2, Akt, and BCL-2 signaling pathways [38,39,40,41], while downregulating tumor suppressor genes such as p53 and PTEN [42,43,44,45]. Here, we analyzed the expression of hENT1 and dCK (proteins involved in Ara-C transport and metabolism), as well as SIRT1, an oncogenic regulator, in the cytarabine-resistant HL60-CR50 cells. We observed a significant decrease in the expression levels of hENT1 and dCK, whereas SIRT1 expression was markedly elevated in chemoresistant cells. Our findings are consistent with previous reports showing that reduced hENT1 and dCK expression, together with increased SIRT1 levels, plays a critical role in the development and maintenance of cytarabine resistance in AML cell lines and patient samples [11,13,21,37,46]. Taken together, these results support the use of HL60-CR50 cells as a robust and reliable cytarabine-resistant model suitable for investigating novel mechanisms and therapeutic strategies aimed at overcoming chemoresistance.

In the present study, we demonstrated that SC, a salt derived from casein (main milk protein) reduces cell viability and enhances apoptosis in chemoresistant cells by downregulating SIRT1 overexpression, while increasing p53 acetylation and cleaved-caspase-3 activation. SIRT1 plays a critical role in promoting tumor cell survival and chemoresistance in various types of leukemia [15,37,47]. Its overexpression deacetylates p53 at lysine 382, thereby suppressing p53-mediated apoptotic responses [16,17,43]. Interestingly, treatment with SC, resulted in a significant decrease in SIRT1 levels in cytarabine-resistant cells, accompanied by a marked increase in p53 acetylation. This modification could enhance the transcription of pro-apoptotic genes such as p21, PUMA, Bax, and PMAIP1, thereby promoting mitochondrial-dependent apoptosis. However, further studies are required to validate this proposed molecular mechanism. These observations are consistent with previous reports demonstrating that several natural products can downregulate SIRT1 overexpression and promote p53 activation, ultimately leading to apoptotic cell death [48,49,50,51]. Romero et al. [15] showed that SC, either alone or combined with antileukemic agents, decreases SIRT1 expression in chemoresistant murine leukemia cells, resulting in reduced proliferation and enhanced apoptosis. Additionally, previous studies have described potential molecular mechanisms through which casein and its derivatives (including SC) may exert antineoplastic effects. These mechanisms include (1) loss of mitochondrial membrane potential, (2) reduced intracellular ATP production, and (3) increased caspase-3 activity, collectively leading to apoptosis [15].

On the other hand, it is well established that suppression of TP53 increases cytarabine resistance in AML patients, leading to poorer prognosis and shorter overall survival [52]. In addition, alterations in apoptosis sensitivity further reduce the likelihood of cell death, thereby increasing the risk of therapeutic failure and relapse [52]. Here, we provide the first evidence, to the best of our knowledge, that SC significantly increases p53 acetylation by downregulating SIRT1, thereby promoting apoptosis in 42% of cytarabine-resistant PML cells, compared with 5% and 8% in the PBS and Ara-C groups, respectively. Moreover, SC treatment induced cleaved-caspase-3 activation, a hallmark of late apoptotic cell death. This observation highlights the relevance of targeting SIRT1, an oncogenic deacetylase that negatively regulates p53 at lysine 382 and contributes to chemoresistance, as a promising strategy to restore apoptotic sensitivity in resistant leukemia cells. Our results are consistent with those reported in the literature, where it has been demonstrated that SC decreases SIRT1 expression and promotes apoptosis in chemoresistant murine AML cells [15]. Similarly, Aguiñiga et al. [13] observed that SC modulates the expression of chemoresistance-associated genes, including ENT1, dCK, and MDR1, and induces apoptosis in chemoresistant murine leukemia cells. Together, these findings indicate that, although cytarabine-resistant PML cells are largely unresponsive to Ara-C, SC exerts a pronounced cytotoxic and pro-apoptotic effect, suggesting a potential therapeutic advantage over conventional chemotherapy.

Although the estimated IC_50_ value for SC is relatively high compared with cytarabine, several findings support its potential translational relevance. Previous studies have shown that SC can stimulate the proliferation of normal healthy bone marrow cells in vitro and in vivo [18,19]. Moreover, in murine models, SC has been reported to activate immune effector cells, particularly macrophages, enhancing the production of pro-inflammatory cytokines such as TNF-α, which contribute to tumor cell apoptosis and immune surveillance [53]. Additional investigations using leukemic mouse models inoculated with WEHI-3 or J774 cells have demonstrated that SC reduces hepatosplenomegaly, decreases solid tumor formation, and improves overall survival [18,54]. Together, these observations suggest that SC may exert beneficial effects on hematopoiesis and immune restoration, which could be clinically relevant despite its higher in vitro IC_50_. However, future in vivo studies in mice inoculated with HL60-CR50 cells are required to determine the efficacy, safety, and therapeutic feasibility of SC—alone or in combination with cytarabine—in overcoming chemoresistance and preventing relapse in PML.

## 4. Materials and Methods

### 4.1. Therapeutic Agents

Cytarabine were obtained from the Pfizer company (New York, NJ, USA), while sodium caseinate was purchased from the Spectrum company (New Brunswick, NJ, USA). Stock solutions prepared in PBS were stored at −20 °C.

### 4.2. Cell Culture

Human acute promyelocytic leukemia HL-60 cells were obtained from the American Type Culture Collection (Manassas, VA, USA). The cytarabine-resistant subline HL60-CR50, derived from HL-60 cells, was generated by continuous exposure to progressively increasing concentrations of cytarabine. Both cell lines were maintained in Iscove’s Modified Dulbecco’s Medium (IMDM; Gibco BRL, *Grand Island, NY,* USA) supplemented with 10% heat-inactivated fetal calf serum (FCS; Gibco BRL, USA), 100 µg/mL streptomycin, and 100 U/mL penicillin (Gibco BRL, USA). Cells were cultured at 37 °C in a humidified atmosphere containing 5% CO_2_.

HL60-CR50 cultures were monitored daily and subcultured at each step using gradually higher Ara-C concentrations until a final concentration of 450 nM was reached. Each escalation in drug dose was implemented only when the proliferation rate of the cells approached that of the parental HL-60 line in the absence of Ara-C.

### 4.3. Cell Proliferation and Viability Assay

Cell viability was determined using the trypan blue exclusion assay (Sigma, St. Louis, MO, USA) in a Neubauer chamber. For cell proliferation assays, both HL-60P and HL60-CR50 cells were seeded at a density of 20,000 cells per well in 96-well plates and incubated for 48 h with increasing concentrations of Ara-C (0, 122.7, 184.1, 306.8, 429.5, and 593.2 nM for HL-60P cells; and 0, 130.9, 531.8, 1084.1, 4340.5, and 8680.9 nM for HL60-CR50 cells). To validate the chemoresistant phenotype, both HL-60 and HL60-CR50 cell lines were treated with the IC_50_ concentration of cytarabine previously determined for parental HL-60 cells (450 nM Ara-C). In addition, HL60-CR50 cells were treated in presence or absence of sodium caseinate (SC) at concentrations of 0, 2, 4, and 8 mg/mL. Following treatment, cell viability was evaluated using the crystal violet assay. Briefly, cells were fixed and stained with crystal violet dye, which was subsequently dissolved in acetic acid. Absorbance was measured at 570 nm using a plate reader (Multiskan GO, Thermo Scientific, Waltham, MA, USA). For the subsequent experiments in HL60-CR50 cells, we used the IC_50_ value of 5.7 mg/mL for SC and the 450 nM Ara-C concentration previously determined in parental cells.

### 4.4. RT-qPCR Assay

The expression of genes related to chemoresistance into the cell were determined by RT-qPCR. Briefly, total RNA was extracted from parental HL-60 and resistant HL60-CR50 cells using TRIzol reagent (Invitrogen, Carlsbad, CA, USA), following the manufacturer’s protocol. RT-qPCR was performed with the QuantiFast^®^ Probe RT-PC kit (Qiagen: Hamburg, Germany) in an Applied Biosystems USA thermocycler with the following conditions: forty amplification cycles of 95 °C for 10 s; 60 °C for 30 s, 72 °C for 15 s. Primer sequences were as follows: hENT1, forward, 5-CTG GAA AGG CGT AGA GGC TG-3; reverse, 5-CTT CCC TTC GCA GAC TGC TT-3; dCK, forward, 5′-AGC AGG GAG TCT GGA GGT AG-3′; reverse, 5′-GAG AAG GCA GAG AAG GCT GG-3′; SIRT1, forward, 5-GCT GGA ACA GGT TGC GGG AA-3′; reverse, 5′-GGG CAC CTA GGA CAT CGA GGA-3′; p53, forward, 5-GTT CCG AGA GCT GAA TGA GG-3′; reverse, 5′-TCT GAG TCA GGC CCT TCT GT-3′ and β-actin, forward 5′-CAC TGT CGA GTC GCG TCC-3′; reverse, 5′-CGC AGC GAT ATC GTC ATC CA-3′. The fold change in the relative mRNA expression of each studied gene was calculated and normalized to the expression of the housekeeping gene β-actin using the double delta CT (2^−ΔΔCT^) method [22].

### 4.5. Immunofluorescence Assay

Parental HL-60 and HL60-CR50 cells were fixed with 4% paraformaldehyde, permeabilized with 0.25% Triton X-100 for 15 min, rinsed with PBS, and blocked with 1% bovine serum albumin (BSA; Sigma-Aldrich, St. Louis, MO, USA) for 1 h. The cells were then incubated overnight at 4 °C with the following primary antibodies: Alexa Fluor^®^ 594-conjugated mouse anti-ENT1 (1:300; cat. no. sc-377283, Santa Cruz Biotechnology, Dallas, TX, USA), Alexa Fluor^®^ 488-conjugated mouse anti-dCK (1:300; cat. no. sc-393099, Santa Cruz Biotechnology, USA), Alexa Fluor^®^ 488-conjugated mouse anti-SIRT1 (1:100; cat. no. 19A7AB4, Abcam, Cambridge, UK), and rabbit anti-cleaved caspase-3 (1:200; cat. no. sc-377283, Abcam, USA). After incubation, the cells were rinsed three times with PBS and subsequently incubated with an Alexa Fluor^®^ 488-conjugated anti-rabbit secondary antibody (1:200; Thermo Fisher Scientific, Waltham, MA, USA) to detect caspase-3. The following day, the cells were rinsed three times with PBS and counterstained with 4′,6-diamidino-2-phenylindole (DAPI; Vector Laboratories, Newark, CA, USA) to visualize the nuclei. Images were acquired using a confocal inverted microscope with a 40× objective (TCS-SP2, Leica, Heidelberg, Germany). For each experimental condition, fluorescence intensity was measured at the level of individual cells within each field. A minimum of 100–120 cells per treatment group were analyzed across three randomly selected fields in three independent experiments. The mean fluorescence intensity for each group was calculated, providing a representative measure of protein expression.

### 4.6. Acetylated p53 Assay

HL60-CR50 cells were treated with or without SC (5.7 mg/mL) or cytarabine (450 nM) for 48 h. Following treatment, cells were collected and washed with PBS. Briefly, 5 × 10^5^ cells were fixed and permeabilized using ice-cold BD Cytofix/Cytoperm™ solution (BD Biosciences, Piscataway, NJ, USA) for 20 min, followed by two washes with 1× BD Perm/Wash™ buffer. Cells were then incubated for 30 min in the dark with an Alexa Fluor^®^ 647-conjugated mouse anti-p53 antibody (BD Phosflow™, Piscataway, NJ, USA), which specifically recognizes acetylation at lysine 382 in the C-terminal domain of p53. After incubation, samples were washed and analyzed on a BD FACSAria™ II flow cytometer (BD Biosciences, Piscataway, NJ, USA).

### 4.7. Apoptosis Assay

HL60-CR50 cells were seeded in 60 mm culture dishes at a density of 5 × 10^5^ cells per dish and incubated for 48 h in the absence or presence of SC or Ara-C. After treatment, cells were harvested and washed with PBS, suspended in 1× binding buffer at 1 × 10^5^ cells/mL, and then stained with annexin V-PE/7-AAD (Becton Dickinson, Franklin Lakes, NJ, USA) following the manufacturer’s instructions. Fluorescence signals for Annexin V-PE and 7-AAD were detected at 528 nm and 650 nm, respectively, using a BD FACSAria™ II flow cytometer (BD Biosciences, San Jose, CA, USA).

### 4.8. Statistical Analysis

All experiments were carried out in triplicate, and results are expressed as mean ± standard deviation (SD). One-way analysis of variance (ANOVA) followed by Tukey’s post hoc test was applied to assess significant differences among multiple groups. For comparisons between two groups, Student’s *t*-test was used. Statistical analyses were performed using GraphPad Prism 8 software. Significance levels are indicated as follows: * *p* < 0.05, ** *p* < 0.01, *** *p* < 0.001, **** *p* < 0.0001; ns, not significant.

## 5. Conclusions

The use of cytarabine-resistant AML cells provides a valuable model for mimicking relapse and resistance to anticancer agents, which are significant contributors to patient mortality. Despite their resistance, these sublines remain sensitive to SC, exhibiting reduced proliferation, increased apoptosis, and elevated levels of acetylated p53 and cleaved caspase-3. These findings underscore the importance of further research into the potential of SC, particularly in combination with cytarabine, to improve treatment outcomes in chemoresistant AML.

## Data Availability

The datasets generated and/or analyzed during the present study are available from the corresponding author upon reasonable request.

## Abbreviations

The following abbreviations are used in this manuscript:

Ara-C	cytarabine
AML	acute myeloid leukemia
SC	sodium caseinate
qRT-PCR	quantitative reverse transcriptase-polymerase chain reaction
ENT1	equilibrative nucleoside transporter 1
dCK	deoxycytidine kinase
SIRT1	transporters silent information regulator 1 homolog 1
P53	tumor suppressor protein
